# Bacterial and Fungal Endophytes: Tiny Giants with Immense Beneficial Potential for Plant Growth and Sustainable Agricultural Productivity

**DOI:** 10.3390/microorganisms7110481

**Published:** 2019-10-23

**Authors:** Olawale Israel Omomowo, Olubukola Oluranti Babalola

**Affiliations:** Food Security and Safety Niche, Faculty of Natural and Agricultural Sciences, North-West University, Private Mail Bag X2046, Mmabatho 2735, South Africa; wale_easy@yahoo.com

**Keywords:** agro-food system crop enhancement, endophytic microbial resources, sustainable agricultural intensification, soil fertility, agro-ecological crop productivity, bio-fertilizers

## Abstract

The conventional means of achieving enhanced agricultural productivity are not ecologically balanced and sustainable. The excessive use of synthetic agrochemicals, declining soil nutrients, and water-use issues, amongst others, are threats to the ecosystem. Additionally, environmental degradation and an increasing global population that will reach 9 billion by 2030 are further considerations. These issues mean a decline in the volume of food resources available to feed the world. Therefore, sustainably increasing agricultural productivity is a necessity for restoring soil fertility, feeding the populace, and improving the ecosystem. A way to achieve this is by using eco-friendly microbial inoculants. Endophytes inhabit the tissues of plants asymptomatically without causing adverse effects. Bacterial and fungal endophytes benefit plants by promoting growth, suppressing pathogens, and improving the stress tolerance and immunity of plants. Despite this vital role played by endophytes in their interactions with host plants, there is still a paucity of relevant review data. More importantly, the prospective use of endophytes as an alternative to synthetic agrochemicals to ensure agro-ecological crop productivity has not been well reviewed in the literature. Therefore, this review sought to highlight the potential use of endophytic microbial resources to achieve enhancements in agro-food system crops in a sustainable manner.

## 1. Introduction

The global community is faced with constraints that adversely affect sustainable and eco-friendly agricultural productivity. Improvements of crop yield and productivity are hampered by numerous limiting factors including poor/erratic rainfall, inadequate water supply and water-use efficiency, and diminishing soil fertility, accompanied by a decline in soil nutrients. These constraints are due, among other factors, to the over-exploitation of agricultural soil, urbanization, land degradation, the excessive use of synthetic agrochemicals, the challenges of destructive phytopathogens and pests, and abiotic stresses [1]. These constraints are exacerbated by the increasing number of people to feed: the human population will number more than 9 billion by the year 2030 [2]. The issue of climatic change and its adverse effects on crop productivity also adds to this herculean task. Even on a smaller scale, changes in local climatic conditions considerably affect plant growth and productivity. Apart from effects resulting from variations in the climatic elements such as radiation, precipitation, humidity, and temperature, the anthropogenic activities of humans are also affecting the climate, which leads to negative consequences for the sustainable productivity of crops in the local ecological system, in the agricultural sector on a larger scale, and even on a global scale [3].

In a bid to overcome these limitations and improve agricultural productivity, modern agricultural production is being strongly intensified through the use of huge quantities of agro-chemicals in the form of synthetic fertilizers and pesticides [4]. Indeed, the conventional means of achieving agri-food security in this era is through the intensification of agricultural production input. This is done by deploying excessive quantities of agrochemicals, which are expensive and pollute the environment, thereby posing a threat to humankind and the ecosystem [5].

Despite the successes recorded with the use of these synthetic agrochemicals, they do have their drawbacks. The excessive use of synthetic agrochemicals has negative impacts on the wellbeing of humans and the proper functioning of the ecosystem. Furthermore, they reduce crop production sustainability [6]. In fact, the intensification of agriculture adversely affects ecological balance, reduces the fertility of the soil, contaminates the food chain, pollutes the groundwater, reduces microbial diversity, reduces soil pH, and leads to increased microbial resistance [7].

To keep up with the increasing population and the attendant challenge of feeding the huge populace, agricultural production needs to be intensified; however, this must be done sustainably. Other alternatives are urgently needed to achieve environmental balance and sustainability. Globally, scientists in the fields of agriculture and the allied sciences are engaging in research to establish innovative means of improving agricultural productivity and sustainability. The intensification of sustainable agriculture is necessary for the achievement of an increase in productivity and maintenance of the ecological balance. This will translate into higher crop yields owing to improvements in plant resilience and the ability of plants to adapt to changing climatic conditions, as well as to biotic and abiotic stress shocks [8].

Improving crop production output in this era, challenged by the enormous constraints that limit agricultural productivity, requires a maximization of productivity, but also that sustainably be concomitantly assured without causing damage to the ecological balance [9,10]. A cheaper, more eco-friendly and sustainable means of achieving agricultural intensification and improving productivity is by adopting the use of microbial inoculants to enhance the availability and use of vital soil nutrients. Furthermore, these inoculants are extremely useful in mitigating both abiotic and biotic stresses in planted crops [11]. 

There has been an upsurge in the use of microbial inoculants in contemporary agricultural production to boost soil fertility and improve the cycling of nutrients, and to enhance growth, vitality, and the productivity of crops [12,13,14]. The use of microbial inoculants, including, bio-fertilizers, biopesticides, bioflocculants, bioremediation agents, and biostimulants, in a sustainable way in order to improve the growth of plants, their disease-resistant properties, and their fitness and vitality is the right way forward. Numerous studies on the beneficial roles of microorganisms that are intimately associated with plants in the rhizosphere, phylloplane, and rhizoplane, such as rhizobacteria that promote plant growth (PGPR), or microorganisms that promote plant growth (PGPM), have been well-documented [15,16,17,18].

Reports highlighting the importance and beneficial properties of microbe-based formulations in enhancing crop development and productivity have been in the news as the basis of the revitalized “greener revolution in agricultural production” [19,20]. Other alternatives are urgently needed to achieve environmental balance and sustainability. Therefore, the focus must be to bring to the forefront simple biological alternatives able to take care of these abiotic and biotic stresses. To effectively combat these challenges, there is a need for novel, trait-specific microbial strains for crop improvement. The application of a single strain does not always lead to positive results. Furthermore, when consortia of beneficial microorganisms are used in plants as inoculants, they confer better results. Moreover, it is imperative to move ahead in this direction because plant infestation by pests and disease-causing pathogens, and the susceptibility of plants to different environmental stress conditions are often due to a reduction in beneficial microbial diversity and an imbalance in the microbiome of the plant-host relationship. The plant microbiome is a fundamental partner in protecting the plant from stresses, in that it synthesizes enzymes or metabolites that can negatively affect plant pathogens; it also produces important phytohormones and ensures the tolerance of the plant to environmental stress [21].

However, lately, the search is now focused on the other important partners of the plant microbiome that colonize the internal tissues of the host plant without causing any disease symptoms—“endophytes”. Microbial endophytes are microorganisms that live in the tissues of a plant without causing any adverse effects to their host plant. These endophytic bacterial and fungi interact with their plant host and elicit positive responses to plant pathogens, herbivore pests, and even to abiotic environmental stresses in their host plants; they also synthesize important bioactive metabolites. Many beneficial traits/functions of their plant host, including the promotion of plant growth, the ability to suppress plant pathogens and to improve the tolerance of the plant to stress, as well as improving the immunity of the plant, have been ascribed to endophytic microorganisms. Also, reports that indicate the potential of bacteria and fungi endophytes for promoting in vitro and in planta growth and for suppressing disease have been confirmed [22,23].

There is no plant in existence that does not have intimately linked endophytes colonizing its tissues [24]. Despite this vital link and the symbiotic role played by endophytes in their host plant, environment, and pathogen interactions, there is still a paucity of review data. More importantly, unlike plant-growth-promoting rhizospheric microorganisms, the prospects of these endophytes as an alternative to synthetic agrochemicals in ensuring sustainable agro-ecological crop productivity have not been well reviewed in the literature.

The focus of this review was to highlight selected research articles published in the web of science that have delved into endophytic bacteria and fungi as potential promoters of plant growth, and as phytopathogens inhibiting bio-inoculants. The mechanisms used in achieving these ends have been elucidated in this article. The present state of knowledge concerning the potential of endophytic bacteria and fungi, as well as the prospective use of these important partners of the plant microbiome as biological inoculants in sustainable crop management, have also been highlighted with regard to smart biotechnological approaches. Furthermore, the need to bioprospect for novel endophytes in different ecological niches using high throughput molecular techniques, to characterize their functions, and to screen and optimize their environmental stress fitness for effective survival and competition in the field, has been reviewed. Hence, this review sought to highlight the important roles that microbial resources play and their potential to enhance the production of agro-food crops in a sustainable and eco-friendly manner.

## 2. Endophytes

The interrelationship of all living things on earth is much more apparent in the plant world. The plant exists in close relationships with the microorganisms in their various ecological niches. The relationship between the plant host and its diverse group of microorganisms is often symbiotic. There are epiphytic, rhizospheric, and endophytic microorganisms that inhabit the different environmental niches of their plant host. Among these different groups of microorganisms that exist in symbiotic association with their plant host, endophytes are the closest and most intimately linked with their plant host. Endophytes can be simply defined as microbial forms that aid in colonizing plant tissues without causing any adverse impacts [25,26]. All living plants are colonized by different endophytes, without any exceptions; indeed, plants are host to a diverse group of endophytic microbes in a mutualistic, beneficial way, which is vital to plant growth and health [27].

Endophytic microbes can enter and colonize plants through the vertical seeding method or through horizontal transmission from the soil to the plants. Either way, mutualistic benefits are still conferred through the plant-microbe interactions [27,28]. This highly beneficial mutualistic interaction makes it possible for endophytic microbes to confer the following benefits on their plant host: they increase the nutrients made available to the plant host, act as protective defenders against pathogens and destructive pests, improve the capability of the plant to withstand or tolerate environmental stresses, help in modulating development of their plant host, and are also helpful in tackling issues related to the growth of unwanted weeds [29,30,31,32]. The traits that the endophytic microbes acquire are considered to be beneficial in that they use different mechanisms to confer these important functions on their host plant.

## 3. Bacterial Endophytes

For plants to effectively thrive in their ecological niche, they form mutualistic interrelationships that are of benefit to the different living organisms in the ecological system. One such positive association is the interaction between microorganisms and the plant [33]. Some tissue-colonizing bacteria form a closely linked association with their host plant and, in fact, grant the plant benefits under both ideal and challenging conditions. 

These endophytic bacteria grant their host plants advantages, which include helping them to mitigate growth limiting biotic and abiotic influences [34]. Endophytic bacteria confer stress tolerance capabilities on their host plant. They induce allelopathic effects in their host plant, while also improving its growth [35]. The functional traits of endophytic bacteria that are highlighted in Table 1 have been associated with the capability of their host plants to thrive and to survive more easily in their respective ecological niches.

Bacterial endophytes have been isolated and identified in diverse plant hosts, environments and in different parts of the plant, including the root tissues, stems, leaves, seeds, fruits, tubers, ovules, and nodules [45]. However, the occurrence of bacterial endophytes is more prevalent in the root tissues as opposed to the aerial plant tissues [46]. Numerous studies that have reported the growth-promoting potency of bacterial endophytes on different crops, including rice, wheat, potato, canola, tomato, and other crops [47,48]. Various investigative studies have alluded to the huge agro-biotechnological potential use of endophytic bacteria as bio-inoculants to achieve a sustainable, eco-friendly, and enduring agricultural production system.

## 4. Fungal Endophytes

Fungal endophytes exist in close, mutually beneficial association with their plant host, in that they provide ecological support to their host plants by allowing them to survive adverse biotic and abiotic stresses. In their turn, the endophytes derive nutrients and protection from the plant [49].

Fungal endophytes colonize plant host tissues such as stems, fruits, flowers, roots, leaves and branches; this is done asymptomatically, without any adverse effects [50]. They constitute a key component in the huge biodiversity of the fungi kingdom. Fungal endophytes are known to confer beneficial effects on their plant hosts. These include the containment or mitigation of damage caused by pests or destructive insects [51,52]. Moreover, reports indicate that plants colonized by these fungal endophytes are less susceptible to the destructive effects of pests. The endophytes confer these benefits on their host plant by interrupting the growth and development phases of the pest; they also affect the feeding pattern and reproductive stages of the pest, thereby affecting its overall survival [53]. Containment of pest damage attributed to fungal endophytes has been highlighted in the case of the maize plant [54]. The control of pest damage has also been reported in the tomato, cotton, and coffee plants, and the banana, faba bean, and common bean plants [55,56]. Other investigators have also reported effective actions of fungal endophytes on the suppression and containment of pest damage in plants [57,58]. The lessening of damage through the containment of pests by the fungal endophytes can be attributed to their production of secondary mycotoxigenic metabolites in their host plants which are toxic to the pests [59].

Another important mutualistic benefit of the endophytic fungal interrelationship with the plant host is the ability of the endophyte to confer both abiotic- and biotic-stress-tolerant traits on its plant host, thereby helping the host to improve its growth and to be less susceptible to disease [60,61]. Fungal endophytes have also proven to be important sources of metabolically active compounds [62,63]. They have the ability to produce vital plant hormonal compounds such as piperine, gibberellic acid, and indole-3-acetic acid, which are required for the promotion of plant growth [62,64]. They are capable of inhibiting plant-disease-causing pathogens, and enable plants to tolerate salinity stress, among other stresses [65,66].

It can be asserted based on studies done in the last few years that endophytic fungi play key functional roles in the ecosystem. Their main effects in terms of beneficial interactions with plants include their ability to make vital growth-promoting nutrients available to the plant; they help suppress noxious plant pests, pathogens, nematodes, and other destructive insects; they help in mitigating environmental stress; and they are also useful in the bioremediation of environmental contaminants. They are able to achieve these effects by using different mechanisms [67,68].

They have also been reported as a potential source of bioactive inoculants that could be useful in achieving agricultural sustainability [69]. Chhipa and Deshmukh [70] reported that endophytic fungi positively influence exudations in the roots of plants, thereby attracting beneficial rhizosphere microbiota that facilitate the transportation of minerals from the soil that are required by plants. They are also beneficial partners in promoting plant immunity and in ecological and physiological adaptations in their plant hosts, thus enabling them to adapt to environmental stresses and to fight against pathogens [67,71]. 

With all the agro-biotechnologically beneficial potential of endophytic bacteria and fungi highlighted, it is not surprising that recent interest in finding safe, environmentally friendly, and sustainable means of improving agricultural out-puts has focused on the implementation of endophytic microbial formulation as an attractive alternative. Fungal endophytes have been proven to be a potential option for attaining sustainable crop intensification owing to their ability to produce vital compounds that promote the growth of plants, inhibit plant-damaging pests and pathogens, and confer immunity fitness and abiotic-stress-tolerant traits on the plant. They could, therefore, be biotechnologically manipulated to improve the productivity and sustainability of agricultural yields (Table 2).

## 5. Entry of Endophytes into the Plant for Colonization and the Mechanisms Involved in Plant Tissue Colonization

In recent times, attention has been focused on the endophytic microbes that inhabit the inner tissues of a plant without causing any adverse effects to their host plant. Numerous reports have been published on the potential use of endophytic microbes as bio-inoculating agents to control plant pathogens, improve immunological fitness of plants and their growth parameters, and to induce functional traits to bolster the tolerance of the plant to abiotic stresses [69,72]. The question that then presents itself is: How do endophytic microbes enter their plant hosts and colonize their tissues?

There are many complexities involved in the entry and eventual colonization of microorganisms into the plant host. The colonization process usually begins with an initial communication signaling preliminary entry of the associated microorganisms and the plant root exudates [46]. The rhizospheric zones of the plant and the root exudates produce vital organic compounds that act as chemo-attractants, which help to facilitate signals or communication between the microbes and plant roots, and then to recruit microbes. This then initiates the process of endophytic entry for the colonization of the tissues of the plant host [81,82].

### 5.1. Post-Preliminary Entry (Adhesion, Attachment) of Endophytes for Plant Tissue Colonization

As stated earlier, communication or signaling takes place between the endophytic microbes and the rhizospheric root exudates of the plant as the preliminary entry step in the endophytic colonization of plant tissue. This step is then followed by the adhesion of the endophytic organism to the surface of the plant host. The next stage in the colonization process is the migration of the endophytes to the plant surfaces as a response to chemo-tactical exchanges between the endophyte and the plant root exudates. This is finally followed by attachment [83]. Attachment is made possible by structural secretory biomolecules/organelles including flagella, pili, fimbriae, lipopolysaccharides, and exopolysaccharides, among others [84,85]. Reports indicate that the initial attachment or colonization by the endophytes of the plant is facilitated by exopolysaccharide metabolites [86,87]. Report further indicate that the initial attachment or colonization is facilitated by lipopolysaccharides [88]. 

The next stage in the complex steps involved in the colonization of plant tissues by endophytes relates to the ways in which endophytes enter the host tissue after establishing their presence on the epiphytic surfaces of a plant. They mainly use passive or active penetration processes to enter the host tissue where cracks open up in the root zones or aerial parts of the plant, such as the stems, flowers, cotyledons, and leaves [89]. Penetration proliferation and attachment have been reported to be mediated by the deployment of bioactive secretory components such as exopolysaccharides, lipopolysaccharides, lytic enzymes (including cellulases), cell-wall-degrading enzymes, and lysozymes, among others [90]. There must be compatibility between the microbial endophyte and the plant host for the colonization of plant tissue to be successful. There must also be effective signaling or communication between the endophytes and the bioactive metabolites of the plant [91,92]. Although the success of colonization depends on diverse factors such as the genotype of the host plant, biotic and abiotic factors, and the extent of nutrient limitations, among other factors, endophytic microbial strains that are efficiently adapted in terms of these factors stand a better chance of success [93].

### 5.2. Transmission of Endophytes (Vertical or Horizontal) for Plant Tissue Colonization

Endophytic bacteria and fungi that are associated with the colonization of plant tissues are transmitted horizontally (plant or soil to plant), vertically (parent plant to seed), or in a mixed way [94]. Most fungal endophytes are vertically transmitted through the seed [95]. Endophytic bacteria, on the other hand, prefers horizontal transmission [96]. Bacterial endophytes are optimally adapted to the horizontal transmission route [32,97], although there have been reports of vertically transmitted seed-borne bacterial endophytes that are of biotechnological importance [98].

Different reports on seed-borne endophytic microbial isolates have been published by various investigators [99,100,101]. The best evidence of endophytic microbes colonizing a plant host through vertical seed-borne transmission has been recorded in research reports that indicate an exchange or overlap in the taxonomy of endophytes in seed plants and their associated seedlings [98,102]. 

## 6. The Potential Use of Endophytic Bacteria and Fungi to Improve Agricultural Productivity

**Figure 1:**
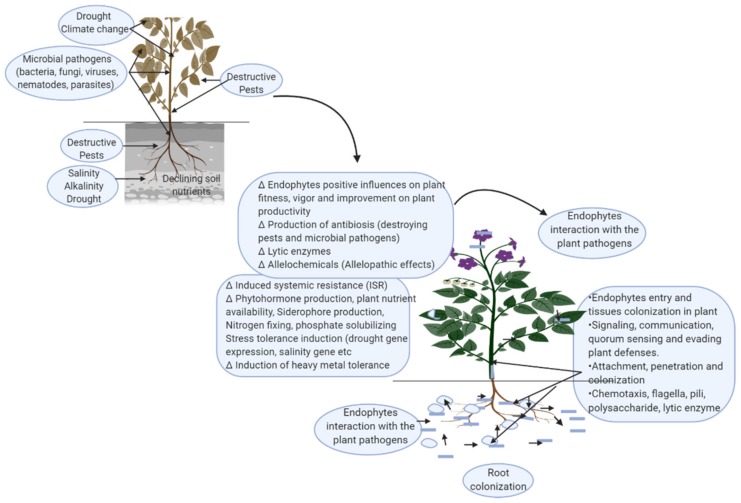
Diagrammatic representation of the functional traits of endophytic bacteria and fungi that are of benefit to plants.

In an era of multi-fold constraints that are adversely affecting improvements in the productivity of agri-food system, there is an urgent need to look beyond these challenges in a modernized, agriculturally sustainable manner in order to maintain environmental balance and achieve sustained productivity from intensified crop production. The potential use of endophytic bacteria and fungi to improve agricultural productivity is a sustainable alternative that has been gaining attention in recent years. Endophytic bacteria and fungi have shown great potential in promoting plant growth (Figure 1), in the biological control of phytopathogens, destructive pests, and insects, in inducing tolerant traits in response to abiotic stresses, and in inducing greater immune fitness in different plants [70,103,104].

The ability of endophytic microorganisms to enter, establish, and firmly colonize plant tissue causes them to present with multi-faceted functional traits that positively influence plant productivity. The question that then arises is: How do endophytes in fact succeed in conferring these beneficial functional traits on plants? Endophytes are a group of ubiquitous and diverse microbes that are found in diverse ecological niches in the tissues of a plant. They inhabit all plants asymptomatically and serve as a treasure trove of biologically important metabolites that could be used for promoting plant growth, potent biocontrol against pathogens and pests, immune defense and fitness, and for conferring functional traits on the plant to allow it to withstand or tolerate external stresses [105,106]. 

A unique property of endophytes is that in their ability to act simultaneously as biocontrol agents and as agents to improve plant growth and yields, they are multifunctional [106,107,108]. Research has shown that endophytic microbes are key players in the interrelationship between them and their plant hosts in the ecological environment, in that they confer functional trait benefits on the host plant. Such benefits include an increased ability to tolerate abiotic stress, to combat or suppress disease-causing pathogens, and to promote the flow of nutrients to the plant and its growth [109]. In their research, Redman, et al. [110] highlighted the fact that endophytes play consequential roles in a plant and also allow for plant growth in marginal areas.

Research has shown that endophytes have functional traits. As mentioned above, they have the ability to increase the nutrient supply to plants, to suppress plant pathogens pests, insects and nematodes—to produce phytohormones in a plant, and to enable plants to tolerate abiotic stresses. Furthermore, they have the ability to fix, solubilize, and mobilize essential elements for the plant to utilize [68,104]. 

Endophytic microorganisms also facilitate improvements in plant productivity by using different mechanisms. Such mechanisms include the breaking down of inorganic nutrient substances from the soil to allow them to enter the roots of the host plant, and the production of enzymes and other essential bioactive metabolites [61]. Apart from these properties, endophytes also improve productivity by protecting their plant host from pathogens in that they regulate the production of important phytohormones, thereby influencing the physiological response of the host plant.

Another key role played by endophytes in improving agricultural productivity is their beneficial interaction with the plant in terms of abiotic stress tolerance. Reports in the literature support the value of endophytes in suppressing abiotic stresses thanks to an array of mechanisms that have been highlighted in different studies [111,112,113,114]. These mechanisms confer abiotic-stress-tolerant capabilities on their plant host. They accomplish this through inducing and expressing genes that are responsive to stress, synthesizing metabolites that act against stress, and also producing scavengers such as reactive oxygen species to take care of free radicals [115].

With the associated adverse effects of climate change on the growth and survival of vulnerable plants, research reports from the literature support the idea that endophytes can help plants to mitigate environmentally induced stresses including drought, higher temperatures, excess salinity, and frequent flooding [116], all of which are classified as abiotic stressors. 

Another important functional role of endophytes in improving agricultural productivity is their ability to resist biotic stresses by suppressing phytopathogens via antagonistic actions [117]. Endophytes have also been reported to inhibit phytopathogens through the expression of genetically linked physiological and defensive pathways in their plant host against disease-causing pathogens and pests [118,119]. 

To effectively inhibit phytopathogens and pests, endophytes produce and secrete bioactive metabolites such as salicylic acid, jasmonic acid, phytoalexins, siderophores, and volatile organic carbon. All of these bioactive metabolites are known for their significant inhibitory actions against phytopathogens and pests that might threaten the plant [120].

## 7. Conclusion and Future Prospects of Endophytes in Sustainable Agricultural Intensification

Bacterial and fungal endophytes have been shown to have great potential as an eco-friendly, natural resource that can be applied in agri-food production to allow for the intensification of crop cultivation and thereby improve levels of agricultural productivity and environmental sustainability. This could be achieved through the sustainable deployment of cheaper, readily available natural bioresources. Current research on endophytic organisms in their roles as potential bioeffectors, bio-fertilizers, biocontrol agents in suppressing biopesticidal threats, and biostimulants has shown that the application of endophytes in the field of agricultural crop production could be worthwhile.

Endophytic organisms encompassing synergetic consortia of both bacterial and fungal endophytes should be developed and supported in their role as bioformulated inoculants for use in agriculture, in order to minimize the use of conventional agrochemicals and thereby ensure the sustainability of the ecological balance. The time to salvage the ecosystem is now. This can be done by engaging in the intensification of sustainable agricultural practices. Endophytic microorganisms have a great role to play in the next green revolution as we attempt to salvage the ecosystem. As greater light is shed on the genetics and the metabolic and physiological processes in endophytic microorganisms, and the symbiotic interrelationships among plants are made clearer through advanced biotechnological screening and investigative processes, these organisms can be used to better effect in field applications as bio-inoculants for the intensification of sustainable agriculture in the near future. 

However, there are still gaps to be filled. Important research directions to pursue in the future include the following:

1. The use of advanced biotechnological tools (omics) to investigate both the community and functionalities of endophytic microorganisms is recommended. A further exploratory investigation into the entire endomicrobiome of plant tissue could lead to the discovery of novel endophytic microorganisms with significant functional traits that could be exploited further in the quest to enhance crops in a sustainable way. Next-generation molecular techniques should be applied to obtain optimum results. It will be essential to use newer biotechnological tools to study the endomicrobiome in terms of genomics, proteomics, and transcriptomic functional traits.
2. There is a need for an extensive bioprospecting study of endophytic microorganisms from diverse ecological niches, (e.g., from extreme environments, the marine environment, etc.,) in order to isolate and characterize novel endophytes with specific traits that could be beneficial to crop production.
3. There is also a need for effective screening for important and essential metabolites that could be deployed directly in the field to circumvent known environmental challenges.

## Figures and Tables

**Table 1:** Plant productivity improvement using bacterial endophytes.

Bacteria Endophyte	Host Plant	Bioactive Influence	References
*Bradyrhizobium sp* SUTNa-2	*Oryza sativa*	Plant-growth-promoting	[36]
*Pantoea dispersa* IAC-BECa-132; *Pseudomonas sp*; *Enterobacter sp*	*Saccharum officinarum*	Plant-growth-promoting	[37]
*Enterobacter cloacae* RCA25; *Herbaspirillum huttiense* RCA24	*Oryza sativa*	Plant-growth-promoting	[38]
*Pseudomonas granadensis* T6; *Rhizobium larrymoorei* E2	*Oryza sativa*	Plant-growth-promoting and pesticide tolerance	[39]
*Bacillus amyloliquefaciens* EPP90; *Bacillus subtilis*; *Bacillus pumilus*	*Pennicetum glaucum*	PGP and abiotic stress tolerance	[40]
*Gordonea terrae*	*Avicena marina*	Plant-growth-promoting	[23]
*Pantoea*, *Pseudomonas*, *Enterobacter*	*Eleusine coracana*	Plant-growth-promoting	[41]
*Bacillus subtilis* LE24, *Bacillus amyloliquefaciens* LE109, *Bacillus tequilensis* PO80	*Citrus spp*	Biocontrol of pathogens	[42]
*Curtobacterium sp* SAK 1	*Glycine max*	PGP and salinity stress tolerance	[43]
*Bacillus tequilensis* (PBE1)	*Solanum lycopersicum*	PGP and biocontrol of pathogens	[44]

**Table 2:** Plant productivity enhancement using fungal endophytes.

Fungal Endophyte	Host Plant	Bioactive Influence	References
*Penicillium aurantiogriseum* 581PDA3; *Alternaria alternate* 581PDA5; *Trichoderma harzianum* 582PDA7	*Triticum aestivum*	Plant-growth-promoting and abiotic stress tolerance	[72]
*Mucor sp*	*Arabidopsis arenosa*	Metal toxicity tolerance	[73]
*Fusarium* sp.	*Dendrobium moniliforme*	Plant-growth-promoting	[74]
*Piriformospora indica*	*Cymbidium aloifolium*	Plant-growth-promoting and abiotic stress tolerance	[75]
*Porostereum spadiceum* AGH786	*Glycine max*	Plant-growth-promoting and salinity stress tolerance	[76]
*Aspergillus awamori* W11	*Withania somnifera*	Plant-growth-promoting	[22]
*Aspergillus fumigatus* TS1; *Fusarium proliferatum* BRL1	*Oxalis corniculata*	Plant-growth-promoting	[77]
*Yarrowia lipolytica*	*Euphorbia milii* L.	Plant-growth-promoting and salinity stress tolerance	[78]
*Aspergillus oryzae*	*Raphanus sativus*	Plant-growth-promoting and biocontrol	[79]
*Paecilomyces variotii*, *Penicillium purpurogenum*	*Caralluma acutangula*	Plant-growth-promoting	[80]
